# O.4.2-7 Feedback from the inside: physical activity effects on interoceptive and emotional awareness

**DOI:** 10.1093/eurpub/ckad133.182

**Published:** 2023-09-11

**Authors:** Noemi Passarello, Fabio Alivernini, Laura Mandolesi, Fabio Lucidi

**Affiliations:** Department of Humanities, University of Naples Federico II, Naples, Italy; Department of Social and Developmental Psychology, Faculty of Medicine and Psychology, University of Roma “Sapienza”, Rome, Italy; noemi.passarello@gmail.com; Department of Humanities, University of Naples Federico II, Naples, Italy; Department of Social and Developmental Psychology, Faculty of Medicine and Psychology, University of Roma “Sapienza”, Rome, Italy; noemi.passarello@gmail.com

## Abstract

**Purpose:**

Interoceptive awareness refers to how one perceives its internal bodily sensations, and it has been associated with the ability to recognize, describe and regulate emotions. Physical activity (PA) has been demonstrated to have tremendous benefits for emotional well-being, but few studies have investigated how PA could moderate interoceptive awareness abilities. In this contribution, we first analysed the association between PA and interoceptive awareness. Then, we tested whether PA could functions as a moderator factor for emotional-bodily awareness relationship.

**Method:**

344 young adults were divided in three experimental groups according to their PA level (sedentary= 60; active= 114; highly active=170), assessed through the International Physical Activity Questionnaire (IPAQ). The Multidimensional Assessment of Interoceptive Awareness (MAIA), and the Toronto Alexythimia Scale (TAS-20) were also administered, to analyse participants’ interoceptive and emotional awareness. Data were analysed through generalized linear model (GLM) (to test PA association with interoceptive awareness), and moderation analysis (to test if PA could moderate bodily-emotional awareness relationship).

**Results:**

GLM outputs showed that MAIA’s interoceptive awareness dimensions were successfully predicted by PA level, specifically Noticing, Not-distracting, Attention-regulation, Emotional awareness, and Self-regulation. Moreover, moderation analysis results showed that PA acted as moderating factor between interoceptive and emotional awareness ().

**Conclusions:**

Despite its preliminary nature, our contribution represents one of the first attempts to operationalize the relationship between Interoceptive and emotional awareness through the inclusion of PA, suggesting the use of practices based on emotional and body awareness to promote psychological and physical well-being as well as successes in sport.

